# Spinal Anesthesia in Genito-Urinary Surgery

**Published:** 1914-12

**Authors:** A. L. Fowler

**Affiliations:** Atlanta, Ga.; Professor of Genito-Urinary Surgery in the Atlanta Medical College; Genito-Urinary Surgeon to the Grady Hospital and St. Joseph Infirmary


### Journal-Record of Medicine

Successor to Atlanta Medical and Surgical Journal, Established 1855
and Southern Medical Record, Established 1870
**OWNED BY THE ATLANTA MEDICAL JOURNAL COMPANY**
PUBLISHED MONTHLY

*Official Organ Fulton County Medical Society, State Examining Board, Presbyterian Hospital, Atlanta, Birmingham and Atlantic Railroad Surgeons' Association, Chattahoochee Valley Medical and Surgical Association, Etc.*

EDGAR BALLENGER, M. D., Editor
BERNARD WOLFF, M. D., Supervising Editor
A. W. STIRLING, M. D., C. M., D. P. H.; J. S. HURT, B. Ph., M.D.
GEO. M. NILES, M. D., W. J. LOVE, M. D., (Ala.); Associate Editors
R. R. DALY, M. D., Associate Editor
E. W. ALLEN, Business Manager

**COLLABORATORS**

W. F. WESTMORELAND, M. D., General Surgery
F. W. McRAE, M. D., Abdominal Surgery
H. F. HARRIS, M. D., Pathology and Bacteriology
E. B. BLOCK, M. D., Diseases of the Nervous System
MICHAEL HOKE, M. D., Orthopedic Surgery
CYRUS W. STRICKLER, M. D., Legal Medicine and Medical Legislation
E. C. DAVIS, A. B., M. D., Obstetrics
E. G. JONES, A. B., M. D., Gynecology
R. T. DORSEY, JR., B. S., M. D., Medicine
L. M. GAINES, A. B., M. D., Internal Medicine
GEO. C. MIZELL, M. D., Diseases of the Stomach and Intestines
L. B. CLARKE, M. D., Pediatrics
EDGAR PAULLIN, M. D., Opsonic Medicine
THEODORE TOEPEL, M. D., Mechano Therapy
A. W. STIRLING, M. D., Etc., Diseases of the Eye, Ear, Nose and Throat
BERNARD WOLFF, M. D., Diseases of the Skin
E. G. BALLENGER, M. D., Diseases of the Genito-Urinary Organs

VOL. LXI ATLANTA, GA., DECEMBER, 1914. No 9

### SPINAL ANESTHESIA IN GENITO-URINARY SURGERY.

BY A. L. FOWLER, M. D., ATLANTA, GA.

PROFESSOR OF GENITO-URINARY SURGERY IN THE ATLANTA MEDICAL COLLEGE; GENITO-URINARY SURGEON TO THE GRADY HOSPITAL AND ST. JOSEPH INFIRMARY.

The object of my appearance before you tonight is to briefly call your attention to spinal anesthesia in genito-urinary surgery, and which is, so far as I have been able to determine, little employed in this section. On the contrary, in other parts of the country and especially in some of the capitals of Europe, spinal anesthesia is used daily, and with very gratifying results.

The principal reason why spinal anesthesia is not more often used is probably due to the fact that in its incipiency ordinary cocain was employed without an adjunct, such as epinephrin or adrenalin chloride. During this stage there followed many failures and an occasional death so that naturally the method rapidly fell into disuse.

Now that the method, through the work of Bier and Donitz, of Berlin, and Freeman Allen, of Boston, has been set upon a safe basis by abolishing the use of cocain and substituting stovain, novocain or tropa-cocain in combination with epinephrin or adrenalin chloride, it is regarded as a remarkably safe one. This is particularly true as regards operations on the lower urinary tract, i. e., from the bladder down; also, in operations about the anus.

In operations in which shock and sepsis will be a feature the method gives such patients the best chance for life. The method is particularly indicated in patients who have diabetes and who would not survive general anesthesia, if such operations as suprapubic cystotomy, enucleation of the prostate, or external urethrotomy were attempted.

Other advantages are that it minimizes postoperative pulmonary complications; reduces the amount of anesthetic to be eliminated by the kidneys; materially lessens the danger of disturbance of the functionating power of the kidney, so that what might ordinarily lead to an anuria is at most not more than an oliguria.

Disadvantages to be occasionally encountered are the failure of the anesthesia; a peculiar type of postoperative shock; migrain, occurring usually by day while the patient is out of bed.

During the past two years I have employed the method in something over thirty cases. I comprehend fully that so few cases are not sufficient to draw conclusions as to the safety of the method notwithstanding I have not lost one. But there are workers who have employed it in three or four hundred cases and more, without a single death, and for this reason the method is worthy of your earnest consideration.

The technique employed in my work is that of Professor Bier, of Berlin, sent to me by Dr. Freeman Allen, anesthetist to the Massachusetts General Hospital, upon the request of Doctor Hugh Cabot. Doctor Armstrong, at the Grady, has administered the anesthetic in the majority of my cases. We have had several failures in securing complete anesthesia, and have attributed these to error in technique, the commonest error being in letting the point of the trocar slip out of the spinal canal at the moment the injection is made. The anesthetic employed has been tropacocain, five per cent. made up in twenty-five per cent adrenalin, and the amount injected has been about fifteen minims. The site of puncture is between the third and fourth lumbar vertebrae. The patients are given a light breakfast on the morning of the operation, sometimes a milk punch or an egg-nog and a quieting dose of morphin before leaving the ward for the anesthetizing room.

The operations in which I have employed this kind of anesthesia have been as follows: Suprapubic cystotomy, enucleation of enlarged prostates, internal and external urethotomies, urethral fistulæ, periurethral abscess with infiltration, varicocele together with curtailment of the scrotum, and epididymotomy. In operating on the urethra and prostate I have gone a step further than simply relying on spinal anesthesia by using in addition a two per cent. solution of cocain instilled into the mucous membrane with a urethral syringe which I think adds greatly to the nerve blocking of this region. I make sure to employ this additional anesthesia if diabetes is a feature.

In the more than thirty cases I have not met with any untoward results except in one case when a patient upon whom I had operated for varicocele, with curtailment of the scrotum, developed on the night of the operation a headache, nausea and pain in his eyes. This was completely controlled by a hypodermic injection of morphin and caffein.

The headache that occasionally occurs is said to be due to a marked increase or decrease in spinal pressure. This headache has been observed after spinal puncture simply for diagnostic purposes, and in some instances perhaps occurs as a result of leakage of spinal fluid through the puncture after withdrawal of the cannula. While I was at Professor Zuckerkandl's clinic, at the Rothchild's 'Spital, in Vienna, where I saw spinal anesthesia used extensively, the practice was to disturb the amount of spinal fluid present in the canal as little as possible. There, the fluid withdrawn through the cannula, was used as the vehicle to make the tropa-cocain solution, the same being reintroduced through the cannula into the spinal canal. But even with this precaution an occasional case would develop a slight headache and may cause nausea. Keyes and MacKenzie report a headache occurring in twenty-five per cent in their series of cases in which they employed stovain instead of tropa-cocain. They state that where they employed tropa-cocain their patients had been remarkably free from headaches. In the New York Medical Journal, November, 1912, these authorities point out the marked immediate rise of temperature so characteristic of perineal section done under general anesthesia; they also present cases to show the absence of temperature when the same operation is done under spinal anesthesia.

I am of the opinion that if these cases had received an instillation of cocain, say two per cent., along the urethra, in addition to the general anesthesia just prior to operation there would not have followed this disturbance of temperatures. Whether I use general anesthesia or spinal anesthesia if I am to operate on the urethra or prostate I always instill cocain along the urethral mucosa a few minutes prior to the operation. This aids greatly in nerve blocking the urethra so that prostato renal and urethro renal inhibitory reflex is reduced to the minimum.

When spinal anesthesia is complete a paralysis of the sphincters occurs so that nothing will be retained per rectum for two or three hours following the operation. It is also to be remembered that care should be had regarding hot water bottle burns about the lower extremities should this form of anesthesia be used.

My chief excuse for this short paper is to emphasize the great value spinal anesthesia has over general anesthesia in cases of diabetes; also, in cases where there are lame kidneys. During the past six months I have removed two prostates from men afflicted with diabetes and they both made uneventful recoveries and are doing well today. And notwithstanding I did the operations in two steps I do not believe they would have survived had I not employed spinal anesthesia.

A great deal of attention and earnest practice seems necessary to administer this form of anesthesia successfully. No matter how competent the surgeon may be reading alone will not qualify him for the work. To become expert one should take lessons under some competent instructor, as for instance one of Bier's assistants, in Berlin, or under one of Dr. Boyer's assistants, at the Hospital Necker, Paris, and in this country under one of the pioneers, Dr. Freeman Allen, at the Massachusetts General Hospital, Boston.

I hope that some one specializing in anesthesia in Atlanta will take such a course so that we can avail ourselves of this important service. About twenty per cent of the cases I have had it administered to have either been only partially anesthetized or have had no anesthesia produced at all when we necessarily had to resort to general anesthesia.

In conclusion let me say that when the anesthesia is complete it is all that can be desired. During the operation we have administered hot broths and brandy to these patients when they were indicated and were relished, and they have gone through the operation without shock. Such patients are returned to the ward in as good condition as when they left it and your other patients awaiting operation see them immediately begin to take nourishment by mouth and naturally make comparisons between this and other forms of anesthesia.

Candler Building, Atlanta.

